# Toward a Better Understanding of the Intention to Use mHealth Apps: Exploratory Study

**DOI:** 10.2196/27021

**Published:** 2021-09-09

**Authors:** Pedro R Palos-Sanchez, Jose Ramon Saura, Miguel Ángel Rios Martin, Mariano Aguayo-Camacho

**Affiliations:** 1 Department of Financial Economy and Operations Management Faculty of Economic and Business Sciences University of Seville Seville Spain; 2 Department of Business Economics Rey Juan Carlos University Madrid Spain

**Keywords:** mHealth apps, mobile apps, eHealth, promotion of health, TAM, PLS–SEM, COVID-19

## Abstract

**Background:**

An increasing number of mobile health (mHealth) apps are becoming available for download and use on mobile devices. Even with the increase in availability and use of mHealth apps, there has still not been a lot of research into understanding the intention to use this kind of apps.

**Objective:**

The purpose of this study was to investigate a technology acceptance model (TAM) that has been specially designed for primary health care applications.

**Methods:**

The proposed model is an extension of the TAM, and was empirically tested using data obtained from a survey of mHealth app users (n=310). The research analyzed 2 additional external factors: promotion of health and health benefits. Data were analyzed with a PLS–SEM software and confirmed that gender moderates the adoption of mHealth apps in Spain. The explanatory capacity (*R^2^* for behavioral intention to use) of the proposed model was 76.4%. Likewise, the relationships of the external constructs of the extended TAM were found to be significant.

**Results:**

The results show the importance of healthy habits developed by using mHealth apps. In addition, communication campaigns for these apps should be aimed at transferring the usefulness of eHealth as an agent for transforming attitudes; additionally, as more health benefits are obtained, ease of use becomes greater. Perceived usefulness (PU; *β*=.415, *t*_0.001;4999_=3.442, *P*=.001), attitude toward using (*β*=.301, *t*_0.01;499_=2.299, *P*=.02), and promotion of health (*β*=.210, *t*_0.05;499_=2.108, *P*=.03) were found to have a statistically significant impact on behavior intention to use eHealth apps (*R^2^*=76.4%). Perceived ease of use (PEOU; *β*=.179, *t*_0.01;499_=2.623, *P*=.009) and PU (*β*=.755, *t*_0.001;499_=12.888, *P*<.001) were found to have a statistically significant impact on attitude toward using (*R^2^*>=78.2%). Furthermore, PEOU (*β*=.203, *t*_0.01;499_=2.810, *P*=.005), health benefits (*β*=.448, *t*_0.001;499_=4.010, *P*<.001), and promotion of health (*β*=.281, *t*_0.01;499_=2.393, *P*=.01) exerted a significant impact on PU (*R^2^*=72.7%). Finally, health benefits (*β*=.640, *t*_0.001;499_=14.948, *P*<.001) had a statistically significant impact on PEOU (*R^2^*=40.9%), while promotion of health (*β*=.865, *t*_0.001;499_=29.943, *P*<.001) significantly influenced health benefits (*R^2^*=74.7%).

**Conclusions:**

mHealth apps could be used to predict the behavior of patients in the face of recommendations to prevent pandemics, such as COVID-19 or SARS, and to track users’ symptoms while they stay at home. Gender is a determining factor that influences the intention to use mHealth apps, so perhaps different interfaces and utilities could be designed according to gender.

## Introduction

### Overview

The use of mobile health (mHealth) apps increased during the first decade of the 21st century [1] and this has led to an increase in the amount of time that users devote to improve their health using mHealth app(s). New ways of monitoring and controlling health indicators and daily activities using new technologies and improvements on the internet have now become available [2].

The increasing use of technology and the internet has forced companies to adapt their marketing strategies to this digital ecosystem. This growth has led to an increase in the use of smartphones around the world [3,4].

For this reason, user behavior and consumption habits with mobile apps have become important fields of research [3,5].

Alharbi et al [6] reported that one type of app which has been increasingly used in recent years is mHealth apps. Support for patients has become more widespread due to the use of these apps. However, users sometimes stop using these apps because they perceive that their usefulness may not cover health quality standards or because the service is not of the same quality as, for example, a visit to the doctor offline [2].

Telemedicine and eHealth have duly become important factors for the analysis, study, improvement, and development of patients’ medical and health care. Electronic health or eHealth was defined by Eysenbach [7] as “health services and information provided by the Internet and related technologies.”

Many mHealth apps provide direct communication links between patients and health care professionals, health education, health portals, wellness management for measuring calories and following a diet, management of diseases such as diabetes and asthma, self-diagnosis to identify symptoms and early diagnosis, medication reminders, and rehabilitation processes and therapies. Therefore, this kind of app could be used to predict what the behavior of patients would be in the face of recommendations to prevent pandemics, such as COVID-19 or SARS, and to track users’ symptoms while they stay at home and follow doctors’ recommendations [8].

The term “application” or “app” refers to a self-contained program or piece of software that is designed to fulfill a particular purpose, and is usually optimized to run on mobile devices, such as smartphones, tablet computers, and wearable devices such as smart watches [3].

Therefore, mHealth apps can improve users’ health by monitoring risks, symptoms, and health care programs. Consumer interest in mHealth apps has increased at the same rate as new technology use in the health care sector. Taking the characteristics shown by mHealth apps into consideration, the technology acceptance model (TAM) was chosen for this study [9].

TAM is a computational system, presented by Davis [10], which analyses users’ decision-making processes when adopting a new technology. The TAM was used in this research paper to investigate the adoption of mHealth apps. External factors that help describe the user adoption of mHealth apps were incorporated into the TAM.

This research therefore fills a gap in the information currently available because it incorporates innovative factors for the adoption of mHealth apps that creators and developers should take into account for successful acceptance and adoption of new mHealth apps. This information duly adds to the existing literature that can be consulted by professionals and researchers.

Therefore, this study addresses the following research question: What factors, including the innovative TAM variables such as promotion of health and health benefits, determine the acceptance of mHealth apps?

This paper is divided into 5 sections. First, the theoretical framework for adoption of mHealth apps is explained. TAM is analyzed and the hypotheses to be studied are formulated. The next section explains the methodology used in the study. The characteristics of the chosen research technique, a survey, are given. This section covers all aspects of questionnaire design and data collection.

Finally, the results of PLS–SEM analysis of the hypotheses and relationships are presented. This section also includes the interpretation, discussion, and implications of the results obtained. The conclusions of the study and the main theoretical and practical implications of the results are also presented.

### Theoretical Background

As stated above, in recent years, researchers have become interested in the adoption of mHealth apps. Research by Housman [11] investigated health information on social media by studying how mHealth apps share results on social media platforms. The increase in use of social networks and the factors that affect the relationship and use of mHealth app were also studied by investigating the social acceptance of mHealth apps by internet user communities [3].

Likewise, Li et al [12] studied emotional bonding of patients with mHealth apps. They showed that users accept this type of apps from an emotional perspective, keeping the disease more in mind and, therefore, applying better monitoring protocols.

Handel [13] studied the use of mobile apps for health and wellness and identified the uses of mHealth apps for health, weight loss, consumption of healthy diet and food, monitoring glucose levels and diabetes, calculating calories consumed, disease diagnosis, meditation, yoga, monitoring sleep quality, and tracking sports activities [14,15]. Therefore, these categories of health care have already been accepted as interesting topics for scientific research in the area of mHealth apps.

Atienza and Patrick [16] studied the acceptance of mHealth apps for the care industry. Furthermore, Grundy et al [17] studied the use of high-quality mHealth apps with innovation-based systems and systematically described the characteristics of recent apps.

Following this line of research, Mueller [18] studied the types of mHealth apps recommended by doctors to their patients, concluding that this type of app is a valid technological support for disease monitoring and treatment.

Likewise, Bloomfield et al [19] studied the influence of SMART goals on the behavior of mHealth app users. Cho [20] investigated the impact of postadoption sentiments on mHealth app use with the postacceptance model and the TAM to find the users’ continued intention to use health apps.

Bort-Roig et al [21] investigated how mHealth apps could improve employees’ sedentary lifestyles while at work and studied the users’ acceptance and continued use of mHealth app. In a similar way, Ashurst and Jones [22] studied the acceptance of mHealth apps among people with diabetes who used one to check and control their condition. It can be seen that the diagnosis and control of medical conditions with technology is an accepted area of scientific research.

Accordingly, Gorkem et al [23] investigated what factors may influence users’ behavioral intentions to adopt and use mHealth apps. To this end, the authors extended the TAM with external factors such as price value, trust factors, and perceived risk and evaluated users’ technology acceptance. The results of this study showed that the first 2 presented a statistical significance with intention to use.

Deng et al [24] studied which determinants influence the adoption of mHealth services among Chinese patients using the TAM extended with trust, perceived risks, and patients’ age and chronic diseases. All external variables were found to be positively correlated with mHealth service adoption.

The study carried out by Mao et al [25] highlighted the importance of studying the recommendations made by patients who have used this type of app to predict what the behavior of patients would be in the face of a change in medical treatment.

In this context, aiming to understand the main advances of mHealth apps, this study takes as a reference the apps regulated by the Food and Drug Administration (FDA). As noted by Humphries et al [2], the FDA is a leading international institution in the regulation of new health products and services and serves as a guide and institutional leader for all other regulatory institutions in the health field around the world, including Spain. Table 1 shows the main mHealth app categories related to this study’s objectives.

**Table 1:** mHealth App categories regulated by the Food and Drug Administration.

Mobile health app (category)	Description	Functions
*Slendertone Connect* (health and wellness)	Allows users to measure physical exercise intensity by connecting an intelligent device.	Measures patients’ resistance, tracks distance traveled, and allows patients to monitor calories.
*Kardia* (medicine)	Allows patients to measure blood glucose levels to detect possible risks and evaluate the patient’s condition.	Tracks patients’ heartbeat, measures the glucose and oxygen level in blood, and shares the patient’s data with the doctor.
*Diasend* (health and fitness)	Measures the patient’s diabetes constants.	Shares data in real time with other users of the app, allows patients to track exercise and calories, and connects patients’ data with other health and medicine apps.
*LibreLink* (medicine)	Checks blood glucose without extracting blood from the finger using a small external device that connects to the app.	Can add notes about food, insulin, and exercise; gives blood glucose readings; and shares information with family, friends, and doctors in real time.
*Qardio heart health* (health and fitness)	Allows patients to control blood pressure, heart rate, and weight. A small external device is used to send the data.	Measurement of blood pressure and patient’s weight, monitoring of heart rate and prediction of heart attacks, and helps share patient information in real time with family and friends.

### Conceptual Framework and Hypothesis Elaboration

The TAM was used to explain the relationship between the acceptance and adoption of technology and the users’ intention to use it [26]. Au and Zafar [27] and Chen and Tan [28] used TAM to demonstrate that perceived usefulness (PU) and perceived ease of use (PEOU) are the most critical factors in the process of adoption and use of new technology. In the TAM, PU and PEOU are considered beliefs and evaluations, respectively, given by users, which influence their attitude toward and intention to use the product (in this case, an app) [29], and finally result in behavior change [30,31].

In the study by Davis [10], TAM was used to explain and predict the use of information systems; in other words, TAM was used to understand the influence of the variables *PU* and *PEOU* on the use of technology. The PU is the belief that a certain technology can improve users’ performance while using it. The PEOU is defined as the degree to which a person believes that using any particular technological system is simple and stress free.

The TAM consistently explains a large part of the variance, 40% according to many authors such as Venkatesh and Davis [32], in the intention to use different information and communication technologies by users in different environments and countries [27,33,34]. Since its appearance, the TAM has been widely analyzed and expanded in different ways [35].

The most important evolutions of TAM have been the TAM2 model by Venkatesh and Davis [32], the Unified Theory of the Acceptance and the Use of Technology by Venkatesh et al [36], the model for the acceptance of technology and user satisfaction by Wixom and Todd [37], and the TAM3 model.

The reasons for choosing the TAM are its tremendous popularity and besides many studies have used the model. The TAM is often considered a common and robust model to address consumer acceptance of an innovative technology [38]. Scherer et al [39] confirmed that the TAM successfully predicts user behavior and can thus be of interest to all potential users of a new technology [29,40,41]. TAM is widely used, with its application extending to a multitude of technologies, especially websites and apps [38].

The TAM has found relevant support in the literature: there are more than 14,870 citations regarding this model within the core collection of the Web of Science database, and more than 51,495 citations have been retrieved from Google Scholar for the article by Davis [10] as of June 2020, 30 years after his first theory.

Therefore, TAM has established itself well as a robust, powerful, and parsimonious model for predicting user acceptance. However, it has been modified through different extensions.

The first of the TAM extensions, the so-called TAM2 [42], is based on the expansion of the PU background. Subsequently, with the same intention as in TAM2, but to complete the model by incorporating the background of the original TAM, Venkatesh and Bala [35] developed the TAM3. More specifically, while TAM2 added the history of PU, TAM3 was expanded into the constructions that precede the PEOU and that were already established in [43,44].

Legris et al [45] made an important critical review of the model and concluded that TAM is useful, but it has to be integrated into a broader one that includes variables related to social and human processes of change.

Similarly, Tang and Chen [46] concluded that current studies on TAM and its extended models have made great progress and recommended paying more attention for future research on new variables that come from other theories or topics that must be introduced in the new model to make it easier to interpret.

Thus, in many health care studies where TAM was applied, the authors have added variables to extend the original TAM to better adapt it to the context of health care [47].

We can find research studies that have used the TAM, such as [48], in which the authors evaluated the acceptance of home telemedicine services by elderly patients. Within the health care domain, the TAM has been used to examine the determinants of adoption or the intention to adopt health technologies [49,50] and to know the effects of cognitive and contingent factors on the health adoption of smartphone apps [51].

The use of computing in the health care field is increasing, but adoption remains a challenge. To understand and introduce the health information technology, a series of behavioral models and innovation acceptance models have been studied and specifically applied the TAM to understand the acceptance of technology [52].

In addition, as in our work, TAM was developed with a focus on technology that can be used voluntarily without the assistance of professional health staff [47].

Furthermore, a recent study in the field of mHealth, in which extended TAM was used [53], indicated that the findings in the literature are contradictory regarding the adoption of mHealth self-monitoring tools, thereby suggesting a gap in the literature that must be covered.

Besides, Thies et al [54] justified that the lack of adoption of a mobile app to support patients in self-management of chronic diseases was mainly due to problems related to the usability of the app and that patients are not comfortable with the technology.

Likewise, Paré et al [55] indicated that people who declare themselves ill are less likely to use digital or traditional tools to monitor their well-being/health than people in good health. Therefore, it is especially important to investigate the adoption of these instruments by consumers considering the characteristics of both the technology and the individuals (users), especially those related to their health [53], as well as the reliability of the model. Extended TAM is decisive in using unused constructs to cover this gap identified in the literature.

The hypotheses below were chosen after reviewing research studies on mHealth apps by Cho [20], Kim and Park [56], and Jeon and Park [57].

Cho [20] and Jeon and Park [57] demonstrated the influence of PEOU on the use of mHealth apps and its effect on PU. Veer et al [58] explained how the intention to use mHealth app influences PU in communities of older people. The following hypothesis was therefore proposed:

H1: Perceived ease of use has a positive influence on perceived usefulness

Veer et al [58], Hu and Bentler [59], and Deng [60] explored the influence and effect of PEOU on attitude toward using. Thompson et al [61] studied the effect of attitude toward using a technology on the intention to use it. Based on their study, the following hypothesis was proposed:

H2: Perceived ease of use has a positive influence on attitude toward use

With the emergence of mHealth, some studies [49,62] confirm the influence of the PU of patients’ intention to adopt a mHealth management service in other cultural contexts [53].

Chauhan and Jaiswal [63] showed that PU influences attitude toward using an mHealth app. The influence of different variables for using different types of mHealth app was also reported. PU demonstrates how a user feels that a particular technology can have a positive effect on his/her life. This influences the user’s attitude toward using the technology [64]. Consequently, the following hypothesis was proposed:

H3: Perceived usefulness has a positive influence on attitude toward use

To investigate PU, Chang et al [65] analyzed the acceptance of a hospital-based eHealth service. The influence of PU on the behavioral intention to use this service by hospital users was also found. Likewise, Klein [66] concluded that PU has a positive effect on behavioral intention to use in his research on patient psychology and the use of eHealth services. Therefore, the following hypothesis was proposed:

H4: Perceived usefulness has a positive influence on behavioral intention to use

Moores [67] concluded that the attitude toward use variable influences the adoption of technological health care services. In addition, Mun et al [68] concluded that behavioral intention to use has a positive effect on the PU of technology by eHealth professionals. From these investigations, the following hypothesis was proposed:

H5: Attitude toward use has a positive influence on behavioral intention to use

Lin and Yang [69] and Buntin et al [70] examined the health benefits of mHealth apps and reported on the main positive health benefits of mHealth apps by applying the TAM for the PEOU construct. Beldad and Hegner [51] studied health benefits with the “health valuation” construct after users tried a fitness app. The confidence that users have in the app was found by extending the TAM with trust, social influence, and health valuation variables. Consequently, the following hypothesis was proposed:

H6: Health benefits have a positive influence on perceived ease of use

Jeon and Park [57] investigated the factors that affect the acceptance of mHealth apps for obesity and found the influence and effect of health benefits on PU. They suggested that more studies should be carried out with the TAM to find out how mHealth apps can help manage and reduce problems with health and chronic diseases [67]. Based on this, the following hypothesis was proposed:

H7: Health benefits have a positive influence on perceived usefulness

Kim and Park [56] improved the TAM with the promotion of health external variable to apply it for evaluating health information technology. Melzner et al [71] studied the influence of mHealth apps on the promotion of health at the workplace and also the attitude of employees toward using an mHealth app. The effects on productivity and health benefits at work upon using an mHealth app were studied by Kelly et al [72]. Ramtohul [73] performed a comprehensive analysis of the decision to adopt eHealth services from the user’s perspective. Therefore, the following hypothesis was proposed:

H8: Promotion of health has a positive effect on the health benefits of mHealth apps

Bert et al [74] studied the influence of mobile phones on promotion of health and concluded that some mHealth apps can help prevent diseases and also influence changes in the users’ health behavior. Ramtohul [73] investigated promotion of health with a construct called “Health Needs,” which expresses the benefits for mHealth app users. Consequently, the following hypothesis was proposed:

H9: Promotion of health has a positive effect on behavioral intention to use an mHealth app

Ramtohul [73] also analyzed the influence of promotion of health on PU in a study on psychological variables. Cho et al [51] analyzed the influence of PU on health benefits for workers who use smartwatch apps [75]. Moores [67] linked the PU of an mHealth app with the promotion of health of app users [8]. Therefore, the following hypothesis was proposed:

H10: Promotion of health has a positive effect on perceived usefulness of an mHealth app

Venkatesh et al [36] pointed out that men and women have different perceptions of usefulness when deciding on technology acceptance. Shabani [76] studied the importance of gender as a moderating variable for adolescents’ emotional health. Bidmon et al [77] indicated that although both men and women use mHealth app, men tend to use it more on mobile devices. Dyck et al [78] studied the moderating effects of age, gender, and education variables and the influence of these on patients’ physical activity. Based on the studies by Venkatesh et al [36] and Shabani [76], the following hypothesis was proposed:

H11: Gender and age moderate all the relationships of constructs in the research model

**Figure 1:**
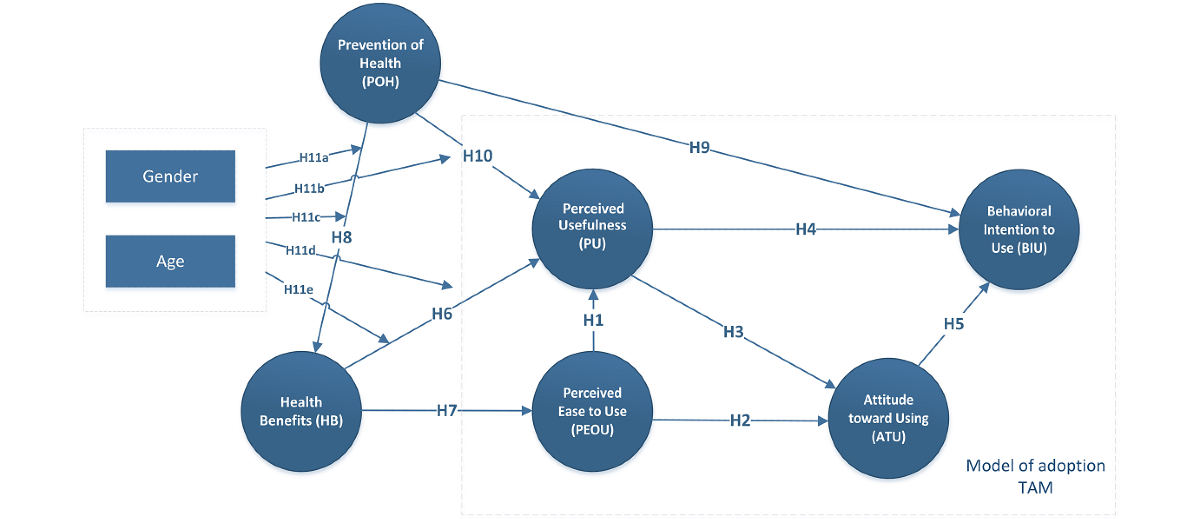
Research model to explore the influence of health benefits and promotion of health on the mHealth app adoption model. TAM: technology acceptance model.

The research model in Figure 1 was formulated to explore the influence of health benefits and promotion of health on the mHealth app adoption model.

## Methods

### Measurement

A questionnaire was created with 24 questions on attitudes and behavior and 5 questions for group classification. The classification questions were for gender, age, job, residence, and education level. The questionnaire was divided into 3 sections. The first section dealt with questions on the users’ behavior, beliefs, and attitudes toward an mHealth app.

Before answering this section and the next one, users could watch a video on different mHealth apps and try them out. A total of 12 different FDA-approved mHealth apps were suggested for trial purposes.

The apps can be found in Google Play or Apple Store by searching their names: my mhealth, Mhealth Medical App, MHealth, Babylon, HealthForYou, Medipal mHealth app, Walking: Pedometer, Medical ID: ICE, Symptom Tracker, ContinuousCare, Medical Record, and ManageMyHealth. All sample members were selected because they indicated that they had previously used mHealth apps and were aware of their functionality and traceability. They were informed about the other apps so that they could take into account additional features of the apps.

The first section of the questionnaire contained 15 questions on PU (n=5), PEOU (n=3), attitude toward using (n=3), and behavioral intention to use (n=4).

The second section consisted of a block of questions on health and disease prevention. These were grouped into health benefits (n=4) and promotion of health (n=4). The last section consisted of 5 questions on the demographic profile of the sample.

Adapted items were used to measure the variables in the TAM [10]. The behavioral items for health were adapted from the studies by Lin and Yang [69] and Jeon and Park [57]. Lin and Yang [69] studied the influence of mHealth app on patients with asthma problems and Jeon and Park [57] studied the influence of mHealth apps on patients with obesity problems. Altogether, there were 24 items in the questionnaire.

All the items, except the demographic profile, were measured using a 5-point Likert scale that ranged from total disagreement (1) to total agreement (5).

A pilot survey was conducted to find the pilot sample’s opinions about the content and structure of the questionnaire, so that the questions could be refined if needed. The pilot survey was conducted on a subsample of 31 individuals whose answers were not added to the final sample.

The subsample followed all the instructions and answered all the questions. Participants were asked to provide comments and suggestions to improve both the instructions and the questions in the questionnaire.

The most important comments were made regarding the items with unclear wording, which were not easily understood, which could cause confusion about the question, or with possible ambiguity in the answers. The wording of these erroneous items was later modified or changed.

The psychometric properties of the proposed scale were then evaluated, along with its ability to identify theoretical concepts and constructs from the data extracted from the questionnaire. The criteria, procedures, and validation techniques for scales proposed by Mackenzie et al [79] were used to create the validation process for the scale used. The measurement model gave satisfactory results.

### Recruitment

The questionnaires were distributed in Spain, both in Madrid and in towns and cities in nearby regions. The prerequisite for the sample was that the user had 4G or Wi-Fi connectivity to the internet. In total, 442 valid questionnaires were collected from the interviewees between January and February 2020.

The sampling was nonprobabilistic and convenient. Google Forms (Google LLC/Alphabet Inc.) was used to prepare the questionnaire, which was then distributed on different social networks, especially LinkedIn (Microsoft).

The SPSS 24 statistical software (IBM) was used to calculate the frequency tables and statistics generated by the sample.

### Demographic Information

The results from the questionnaires showed that 242/442 members (54.8%) were men, 195/442 (44.1%) were women, and 5/442 were others (1.1%).

Of these, 186 participants live in small populations of less than 5000 inhabitants (42.1%), which makes the sample interesting, as getting to hospitals and health centers may be difficult for them. Furthermore, 336 participants were aged between 18 and 30 years (76.0%) and 291 had studied at a university (65.9%); 64.9% (n=287) of the sample were students.

### Statistical Analysis

Data analysis and hypothesis testing were carried out using structural equation modeling (SEM) with variance, which allowed for a statistical examination of the interrelated dependency relationships between the latent variables and the indicator variables of the research model by directly measuring observable variables [80].

SEM was used together with partial least squares (PLS). PLS trajectory modeling can be understood as a complete SEM method to study composite factor models by measuring constructs, estimating structural models, and performing model fitting tests [81].

The PLS–SEM statistical analysis technique, based on the structural equation model, was used, as it is especially recommended for exploratory research. It allows the modeling of latent constructs with both formative and reflective indicators to analyze the collected data [82]. In addition, PLS is appropriate for the prediction and analysis of relatively new phenomena [83]. The SmartPLS 3 software (SmartPLS GmbH) was used in this study [84].

Reinartz et al [85] investigated the conditions under which PLS–SEM should be used in research analysis, and concluded that the technique can be applied for a relatively new object of research with a model that is not fully consolidated. As these were the conditions in this research, we chose to use PLS–SEM. Besides, ours is an exploratory approach [86] for which this type of data analysis is highly recommended [87].

The PLS–SEM technique was also used because one of the aims of this research was to check whether the model was predictive. Chin and Newsted [83], Fornell and Larcker [88], and Hair et al [89] had already shown that PLS–SEM can be used for this purpose.

Fornell and Bookstein [90] state that PLS explicitly defines the latent variables, constructs, or combinations, which can easily be measured. The use of these factors is another point that justifies the use of SEM, as shown in similar studies by Sarstedt et al [80], Henseler [91], and Rigdon et al [92].

Based on the research studies by Sarstedt et al [80], Hair et al [89], and Cepeda-Carrion et al [93], the choice of the best SEM approach depends on the type of latent variables being measured, with the aforesaid studies recommending PLS for reflective or common factor constructs. The information required to analyze these factors was found from other related variables, which is another condition for which PLS–SEM is recommended [80]. Investigation and adoption of mHealth apps is a recent area of research. Because this study is exploratory, PLS–SEM is recommended.

The Harman single-factor test was used as an indicator in the subsequent common method bias test [94,95]. Using this test, no single factor was detected that could explain most of the total variance, which suggests that it is very unlikely that any selection bias exists.

## Results

### Measurement Model

The measurement model was tested for internal reliability, convergent validity, and discriminant validity. The internal reliability was evaluated using Cronbach *α* which needs a value of at least .70 for acceptable internal consistency [96]. Causality was analyzed using indicator loadings. Composite reliability was also used to investigate causality [97]. All the constructs had internal consistency, as their Cronbach *α* values were higher than .7 [86,88,98]. To assess convergent validity, Fornell and Larcker [88] used the average variance extracted (AVE) method and stated that an acceptable value for this factor is 0.50 or more.

The structural model was then analyzed using a bootstrapping technique configured to readjust 5000 subsamples to estimate the statistical significance of the path coefficients [99].

Table 2 shows the element loads, Cronbach *α*, and AVE which were found for the constructs. Cronbach *α* values ranged from .899 to .789, which is higher than the recommended level of .70, and therefore indicates strong internal reliability for the constructs. The composite reliability ranged between 0.930 and 0.877 and the AVE between 0.651 and 0.783, which are higher than the recommended levels. The conditions for convergent validity were therefore met. The discriminant validity was calculated with the square root of the AVE and the cross-loading matrix. For satisfactory discriminant validity, the square root of the AVE of a construct should be greater than the correlation with other constructs [88].

These researchers carried out simulation studies to demonstrate that a lack of discriminant validity is better detected by means of another technique, the heterotrait-monotrait ratio, which they had discovered earlier. All the heterotrait-monotrait ratios for each pair of factors was less than 0.90.

**Table 2:** Reliability, validity of the constructs, Fornell–Larcker criterion, and HTMT.

Construct	Cronbach *α* alpha	CR^a^	AVE^b^	Fornell-Larcker Criterion							HTMT^c^					
				ATU^d^	HB^e^	BIU^f^	PEOU^g^	POH^h^	PU^i^	ATU		HB	BIU	PEOU	POH	
ATU	.861	0.915	0.783	0.898												
HB	.899	0.930	0.768	0.703	0.867					0.828						
BIU	.883	0.920	0.742	0.743	0.711	0.887				0.777		0.788				
PEOU	.789	0.877	0.703	0.596	0.555	0.556	0.854			0.682		0.663	0.641			
POH	.844	0.906	0.762	0.722	0.814	0.719	0.544	0.851		0.788		0.776	0.865	0.632		
PU	.866	0.903	0.651	0.771	0.740	0.771	0.576	0.728	0.811	0.872		0.844	0.822	0.668	0.795	

^a^CR: composite reliability.

^b^AVE: average variance extracted.

^c^HTMT: heterotrait-monotrait.

^d^ATU: attitude toward using.

^e^HB: health benefits.

^f^BIU: behavioral intention to use.

^g^PEOU: perceived ease of use.

^h^POH: promotion of health.

^i^PU: perceived usefulness.

### Structural Model

In this next stage, the proposed model was analyzed in detail. The structural model was built up from the different relationships between the constructs. The hypotheses for the study were tested by analyzing the relationships between the different constructs in the model to see if they were supported [83,85,100].

The assessment of the significance of structural model is usually preceded by performing an analysis of the indicator reliability and the internal consistency reliability to prove the lack of multicollinearity. The variance inflation factor values obtained were less than 5 and ranged from 1.603 (PEOU3) to 3.496 (behavioral intention to use 3).

**Figure 2:**
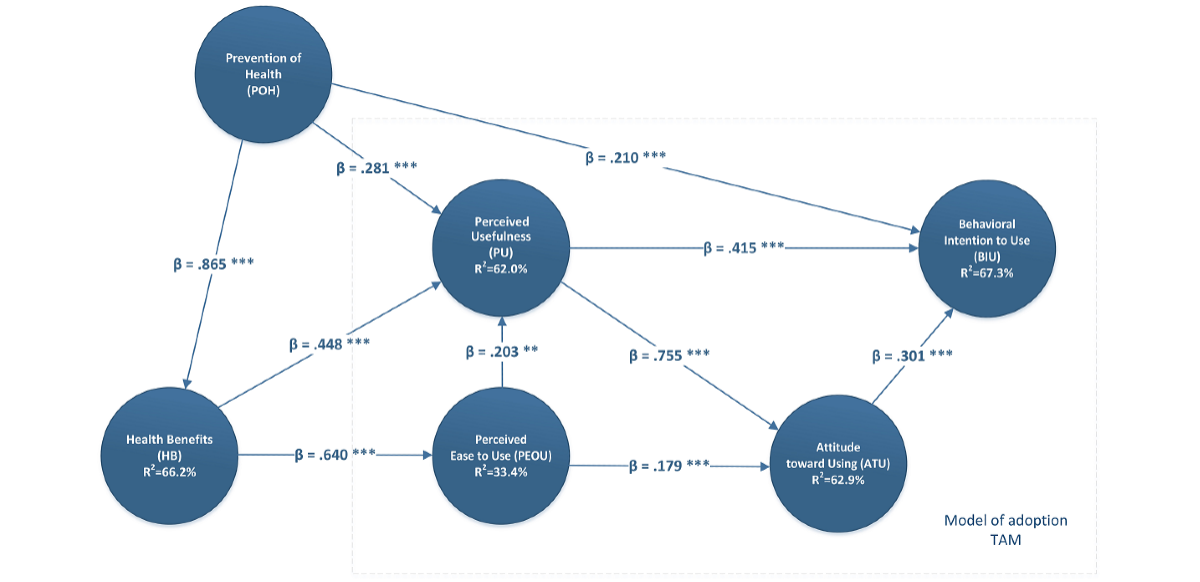
Analysis results (path coefficient, *β*, and *t* statistic are presented). TAM: technology acceptance model.

The variance is found from the values for the reflective indicators given by the constructs [101,102]. This was found numerically by calculating the *R^2^* values, which are a measure of the amount of variance for the construct in the model. The bootstrap method was used to test the hypotheses. The detailed results (path coefficient, *β*, and *t* statistic) are summarized in Table 3 and Figure 2.

PEOU is positively associated with PU (*β*=.203, *t*_0.01;499_=2.810, *P*=.005) and attitude toward using (*β*=.179, *t*_0.01;499_=2.623, *P*=.009), and therefore, H1 and H2 were compatible with the proposed model with a 99% level of confidence.

Likewise, PU, another relationship established in the TAM, positively influenced the variable attitude toward using. This relationship was therefore confirmed and was compatible with the proposed model (*β*=.755, *t*_0.001;499_=12.888, *P*<.001) with a high level of confidence (99.9%).

The TAM constructs that influence behavioral intention to use, such as PU (*β*=.415, *t*_0.001;4999_=3.442, *P*=.001), have a significant influence on the intention to use an mHealth app. Therefore, H4 was supported for the proposed model with a confidence level of 99.9%.

The results also indicated that the research model explains 76.4% of the variance of the intention to use an mHealth app (*R^2^* for behavioral intention to use=76.4%, *R^2^* values for attitude toward using, health benefits, PEOU, and PU are 78.2%, 74.7%, 40.9%, and 72.7%, respectively). The result of a single linear regression from attitude toward using mHealth apps and behavioral intention to use confirmed that attitude toward using is positively associated with behavioral intention to use an mHealth app (*β*=.301, *t*_0.01;499_=2.299, *P*=.02). This means that H5 was supported (99%).

The hypotheses for the external variable health benefits of the original TAM were all supported with the same level of confidence (99.9%). Therefore, the health benefits variable was shown to have a significant influence on PU (*β*=.448, *t*_0.001;499_=4.010, *P*<.001) and therefore H6 was supported.

Likewise, health benefits also positively influenced PEOU (*β*=.640, *t*_0.001;499_=14.948, *P*<.001), which shows that H7 was supported. The other external variable (ie, promotion of health) was found to significantly influence health benefits (*β*=.865, *t*_0.001;499_=29.943, *P*<.001), which means that H8 is supported with the highest values in this research model (99.9%). H7 and H8 had the highest *t* statistic value of all the studied hypotheses (Table 3).

H9 and H10 studied the association of promotion of health with behavioral intention to use (*β*=.210, *t*_0.05;499_=2.108, *P*=.03) and PU (*β*=.281, *t*_0.01;499_=2.393, *P*=.01) with a 99% level of confidence. H9 had the lower *t* statistic value of all the studied hypotheses (95%).

**Table 3:** Results of hypothesis: path coefficients and statistical significance (n=5000 subsamples).^a^

Hypothesis	*β* (coefficient path)	*t* statistic	*P* value	Supported
H1: Perceived ease of use → Perceived usefulness	.203	2.810	.005	Yes^b^
H2: Perceived ease of use → Attitude toward using	.179	2.623	.009	Yes^b^
H3: Perceived usefulness → Attitude toward using	.755	12.888	<.001	Yes^c^
H4: Perceived usefulness → Behavioral intention to use	.415	3.442	.001	Yes^c^
H5: Attitude toward using → Behavioral intention to use	.301	2.299	.02	Yes^b^
H6: Health benefits → Perceived usefulness	.448	4.010	<.001	Yes^c^
H7: Health benefits → Perceived ease of use	.640	14.948	<.001	Yes^c^
H8: Promotion of health → Health benefits	.865	29.943	<.001	Yes^c^
H9: Promotion of health → Behavioral intention to use	.210	2.108	.03	Yes^d^
H10: Promotion Of Health → Perceived usefulness	.281	2.393	.01	Yes^b^

^a^For 5000 subsamples, we used a *t* distribution (4999) of students in single queue.

^b^*P*<.01 (*t*_0.01;499_=2.333843952).

^c^*P*<.001 (*t*_0.001;499_=3.106644601).

^d^*P*<.05 (*t*_0.05;499_=1.64791345).

The measurements for approximate adjustments of the model [81,91] are given by the standardized root mean square residual (SRMR) value [103], which measures the difference between the observed correlation matrix and the implied correlation matrix of the model. SRMR shows the average magnitude of these differences.

A low value of SRMR means that the fit is better. In our case SRMR=0.023, which was within the recommendations for a model with a good fit. A good fit is considered to be shown with an SRMR value of less than 0.08 [103].

Regarding the evaluation of the overall fit of the model, Benitez et al [104] recommend evaluating a saturated structural model by investigating discrepancy between empirical and model-implied indicator variance–covariance matrix. Bootstrapping results show that the SRMR sample mean for the saturated model (0.023) is below the 95% mark of its corresponding reference distribution (0.027).

The blindfolding procedure omits part of the data for a given construct during the estimation of parameters. Estimated parameters are then used to try to recreate the omitted data [101]. It is possible to study the predictive relevance of the model in this way using the Stone–Geisser (*Q^2^*) test [105,106]. This test revealed that the model has predictive capability. As can be seen in Table 4, all endogenous constructs fulfill *Q^2^* > 0. Values of 0.02, 0.15, and 0.35 for *Q^2^* in the Stone–Geisser test indicate small, medium, and great predictive relevance [107].

As per the *R^2^* (see Table 4 and Figure 2) values reported by Chin [101], we conclude the following: If *R^2^*=0.67, the result is considered substantial; 0.33, the result is considered moderate, and 0.19, the result is considered weak. The *R^2^* obtained for the main dependent variable of the model, behavioral intention to use, was 76.4%

This value shows that this model is “substantially” applicable for the adoption of an mHealth app. The variables that are not endogenous do not have a value for *R^2^*.

The blindfolding technique consists in omitting part of the data for a given construct during the estimation of parameters, and then trying to estimate what was omitted from the estimated parameters [83].

In this way the predictive relevance of the model was studied and using the Stone–Geisser (*Q^2^*) test the model was shown to have predictive capacity [105].

Therefore, all constructs, except PEOU, in the studied model have great predictive relevance, as the values of *Q^2^* are greater than 0.35 (Table 4). The proposed research model thus has good predictive power when explaining behavioral intention to use an mHealth app.

Effect size shows the strength of the relationship between 2 variables in the research model on a numeric scale. The effect size (*f*^2^) shows how much an exogenous latent variable contributes to the *R^2^* value of an endogenous latent variable. The *f*^2^ values 0.02, 0.15, and 0.35 indicate small, medium, and large effect size [100]. Cohen’s tables [107] showed that for 95.2% statistical power and an average effect size of *f*^2^=0.15, a minimum of 107 questionnaires would be needed. In our case the number of samples was 442, showing that this research has adequate statistical power.

**Table 4:** *R^2^* and *Q^2^* results.

Construct	*Q^2^*	*R^2^* (%)
Attitude toward using	0.478	78.2
Health benefits	0.465	74.7
Behavioral intention to use	0.491	76.4
Perceived ease to use	0.229	40.9
Promotion of health	N/A^a^	N/A
Perceived usefulness	0.381	72.7

^a^N/A: not applicable.

### PLS–SEM Results With Moderator (Gender and Age)

In order to check H11 and measure the potential moderating influence of gender and age, we performed a multigroup analysis [108].

First, the sample was divided by gender into men and women. The following process was then repeated, dividing members of the sample into old and young people.

However, before doing this test it is necessary to analyze the measurement invariance of the composite models (MICOM) technique [80]. This test will ensure that the effect of gender is restricted to the trajectory coefficients of the structural model and not to the parameters of the measurement model [109]. As described in Tables 5 and 6, we find the invariance of the measurement in the case of gender, but not in the case of age (Table 6) for the variables attitude toward using, health benefits, behavioral intention to use, perceived ease to use (PEOU), promotion of health, and PU.

**Table 5:** Results of the measurement invariance of composite models (MICOM) procedure (gender).

Construct	Step 1	Step 2			Step 3a					Step 3b					
	Configural invariance	Compositional invariance			Equal variances					Mean original difference (men–women)		Equal means			
		Original correlation	5%	Partial measurement invariance established	Variance original difference (men–women)	2.5%	97.5%	Equal			2.5%		97.5%	Equal	
ATU^a^	Yes	1.000	1.000	Yes	0.209	–0.182	0.182	No	–0.070		–0.308		0.303	Yes	
BIU^b^	Yes	1.000	1.000	Yes	–0.011	–0.211	0.176	Yes	0.234		–0.278		0.265	Yes	
HB^c^	Yes	1.000	1.000	Yes	0.061	–0.197	0.185	Yes	0.146		–0.277		0.282	Yes	
PEOU^d^	Yes	1.000	0.998	Yes	–0.054	–0.202	0.187	Yes	0.095		–0.267		0.270	Yes	
POH^e^	Yes	1.000	1.000	Yes	0.092	–0.189	0.167	Yes	0.242		–0.288		0.279	Yes	
PU^f^	Yes	.999	0.999	No	0.152	–0.198	0.174	Yes	–0.123		–0.287		0.265	Yes	

^a^ATU: attitude toward using.

^b^BIU: behavioral intention to use.

^c^HB: health benefits.

^d^PEOU: perceived ease of use.

^e^POH: promotion of health.

^f^PU: perceived usefulness.

**Table 6:** Results of the measurement invariance of composite models (MICOM) procedure (age).

Construct	Step 1	Step 2				Step 3a					Step 3b				
	Configural invariance	Compositional invariance				Equal variances					Mean original difference (young people–old people)		Equal means		
		Original correlation	5%	Partial measurement invariance established	Variance original difference (young people–old people)		2.5%	97.5%	Equal			2.5%		97.5%	Equal
ATU^a^	Yes	1.000	0.999	Yes	–0.399		–0.298	0.307	No	–0.665		–0.398		0.537	No
BIU^b^	Yes	1.000	0.999	Yes	–0.520		–0.298	0.298	No	–0.590		–0.423		0.538	No
HB^c^	Yes	1.000	0.998	Yes	–0.461		–0.300	0.304	No	–0.624		–0.410		0.538	No
PEOU^d^	Yes	0.999	0.993	Yes	–0.324		–0.315	0.298	No	–0.432		–0.400		0.512	Yes
POH^e^	Yes	1.000	0.998	Yes	–0.470		–0.299	0.298	No	–0.239		–0.419		0.539	Yes
PU^f^	Yes	1.000	0.997	Yes	–0.606		–0.312	0.293	No	–0.360		–0.401		0.500	Yes

^a^ATU: attitude toward using.

^b^BIU: behavioral intention to use.

^c^HB: health benefits.

^d^PEOU: perceived ease of use.

^e^POH: promotion of health.

^f^PU: perceived usefulness.

## Discussion

### Principal Findings

The results of this study confirmed that the variable that has the strongest impact on the behavioral intention to use of mHealth apps in Spain is PU. This variable also has a very high predictive capacity as its determination coefficient is high [81,108]. The next most important variable in the model is health benefits.

The results of this research could be applicable to other EU countries with similar levels of internet access. However, it must be taken into account that most of the participants lived in areas with less than 5000 inhabitants (186/442 participants, 42.1%), where acceptance of mHealth apps is also determined by the close social environment. In this type of environment, users of mHealth apps can offer an effective short-term consultation for families and acquaintances before they make a decision to visit hospitals or health clinics.

### Comparison With Prior Work

These findings are consistent with previous studies on PU for the acceptance of medical information systems [57,69,101]. These studies also found that PU significantly influences the adoption of medical information systems.

Promotion of health was also found to have a significant effect on health benefits of using mHealth apps in this study, as mHealth apps positively promote and improve the health of mHealth app users in Spain. This relationship was the strongest among all the relationships studied in this research and shows the usefulness of mHealth apps for improving health.

This is important when promoting the idea of preventing diseases and other ailments with mHealth apps, such as controlling continued physical exercise, consumption of certain foods, monitoring the evolution of potential and current patients, and using smartphones or tablet PCs to help prevent health problems. These results are consistent with the findings from a previous study [110].

H8 has been revealed as the relationship with the greatest burden and confirms the extraordinary influence it has on health benefits (*β*=.865, *t*_0.001;499_=29.943, *P*<.001). This means that eHealth apps that take care of nutrition, improve sports activity, or make mealtimes more respectful are perceived by respondents as favoring aspects related to blood pressure, weight loss, blood sugar levels, or mood. In other words, users consider that apps related to healthy habits should be developed. This means that H8 is the most reliable and significant relationship among all.

The second hypothesis with the greatest burden and influence was H7. The relationship between health habits and PEOU of apps indicates that the more beneficial the eHealth app is, the easier it should be to use. Furthermore, the third hypothesis with the greatest intensity is H3, which shows that the perception of usefulness of an eHealth app has an extraordinary influence on the attitude of use. This means that the selling strategy of these apps must be aimed at transferring 2 very important aspects to the user: on the one hand, the usefulness of eHealth as an attitude transforming agent, and on the other, the more health benefits are obtained, the easier it is to use. In addition, these 3 relationships (ie, H3, H7, and H8) were very significant (99.9%).

The TAM is applicable to the use of eHealth apps as was the case with other studies, but with the influence of the “health promotion” and “health benefits” constructs. In addition, health promotion is directly related to the main dependent variable in the behavioral intention to use model. Therefore, health promotion is a construct that should be considered in future research, as it is also directly related to the final construct of the behavioral intention to use as well as indirectly to the PU.

In this study it was demonstrated that mHealth apps were easy to use and that users were familiar with the basic functions and applications of the internet. This is justified by the fact that health benefits had a very significant influence on the perceived usability (PEOU). This is an important point to highlight when explaining mHealth apps, as this can help ensure that mHealth apps are used as often as necessary to achieve effective results. However, the influence of PEOU on PU is the relationship with the lowest load among all (*β*=.179, *t*_0.01;499_=2.623, *P*=.009) and a 99% confidence level. Likewise, the PEOU has a moderate variance (*R^2^*=40.9%), which is why a moderately atypical result was obtained in this research. PEOU has a positive relationship with PU, which suggests that users will not need to learn new skills to use mHealth apps. The sample in this study, however, did not consider it an important factor in this model. In all probability, the advancement of usability of smartphone interfaces reduces the influence of PEOU, so people might need to use smartphones to be able to use these types of apps [111]. These results could be explained by the fact that the Spanish population is already familiar with health promotion and also that current mHealth apps are easy to use and accessible.

The remaining endogenous variables had a very high explanatory capacity (>70%). This gives the model a great capacity to explain the reality of the users’ behavior before using eHealth apps, as in the case of behavioral intention to use it was 76.4%.

The results obtained for the relationship between the PEOU and the attitude toward use predict a smooth learning curve. This suggests that the adoption of mHealth app will be permanent and stable in the future. The use of mHealth apps will not present any significant difficulties that may cause users to abandon it.

Our study also confirmed that health promotion has a positive influence on behavioral intention to use and perception of usefulness (PU). In both cases, the level of trust is high, which shows that health promotion is an important factor in this model. Health promotion was also found to have an indirect influence on health benefits. This result supports the previously reported finding that app titles influence behavioral intention to use [112]. Specifically, we found that apps with titles related to symptoms have a significantly lower number of installs as compared with those whose titles are not related to symptoms.

Finally, a moderating capacity was found with a 95% confidence level regarding gender. We found that the 2 relationships with the lowest level of confidence in the model (Table 7), H1 or the relationship of the perception of ease of use with PU (*β*=–.422, *P*=.015) and H9 or the relationship of health promotion with behavioral intention of use (*β*=–.239, *P*=.04), show significant differences between men and women. Furthermore, gender-moderated behaviors were found in H10, indicating that health promotion also influences the perception of usefulness differently according to gender (*β*=.178, *P*=.01).

**Table 7:** PLS^a^–SEM^b^ results with moderator (gender).

Hypothesis	*β* (Coefficient path)	*P* value	Support
H1: Perceived ease of use → Perceived usefulness	–.422	.01	Yes^c^
H2: Perceived ease of use → Attitude toward using	–.100	.24	No
H3: Perceived usefulness → Attitude toward using	.318	.18	No
H4: Perceived usefulness → Behavioral intention to use	.166	.21	No
H5: Attitude toward using → Behavioral intention to use	–.107	.49	No
H6: Health benefits → Perceived usefulness	.266	.11	No
H7: Health benefits → Perceived ease of use	.003	.94	No
H8: Promotion of health → Health benefits	–.318	.22	No
H9: Promotion of health → Behavioral intention to use	–.239	.04	Yes^c^
H10: Promotion of health → Perceived usefulness	.178	.01	Yes^c^

^a^PLS: partial least squares.

^b^SEM: structural equation modeling.

^c^For 500 subsamples, we used a *t* distribution (4999) of students in a single queue: *P*<.05 (*t*_0.05;4999_=1.64791345).

The other moderating variable (ie, age) was not supported, coinciding with the results of similar studies [113].

Therefore, mHealth app is an effective way to promote good health and habits in the population. Participants in the study believed that mHealth apps could help them improve their health, maintain a meal schedule, take part in more sporting activities, or improve the hours slept at night. Thus, mHealth apps can promote healthy habits and improve the users’ quality of life.

### Conclusions

#### Theoretical Implications

As has often been addressed in previous mHealth studies [114,115], health apps on smartphones can serve as very realistic health care alternatives, helping people save on medical expenses and being more effective in managing their personal health. Therefore, we agree with a previous work [20] that the potential advantages of using health apps (mHealth) in terms of improving overall health can be harmed without the use of apps.

The extended TAM adoption model was found to be fully valid for the study of mHealth app use and acceptance in Spain. This result could be extrapolated to other EU countries with similar levels of internet accessibility and sociodemographic characteristics.

This study identified the variables that influence people’s intention to use mHealth apps. Using an extended TAM, PU was found to be the most significant variable influencing adoption of mHealth apps in Spain. This means that the most important factor for users are the ways in which mHealth apps can help them. This result is important because users of this type of apps must first understand the utility of the use of these apps, so that they can become cognizant about how they can improve treatment of their diseases and their control.

#### Practical Implications

Other external variables, such as promotion of health, have a significant effect on the health benefits of mHealth app use. This result showed that users consider maintenance or improvement of health as an additional health benefit provided by these apps.

The predictive capacity of the model and the predictive capacity can be very useful in preventing diseases that need controlled habits. Examples are indulging in regular physical exercise; consumption of certain foods; monitoring the evolution of current and potential patients; and using smartphones, tablets, and other medical devices to prevent health problems. Besides, care centers should have Wi-Fi access so that patients can carry out real-time diagnostic tests.

The results of this research show that gender is neither completely decisive nor moderating in the behavioral intention to use mHealth apps. This means that adoption of mHealth apps for promotion of health was moderated only by gender. Another important factor influencing mHealth app use is PEOU.

Therefore, user-friendliness and health promotion should be gender sensitive when applying utilities to apps. Accordingly, app developers should take into account users’ gender and introduce some changes in usage and health promotion levels.

The results obtained using the extended TAM show that promotion of health and health benefits are important variables for mHealth apps users because they indirectly influence the adoption of the technology. This means that mHealth apps could be an alternative way to promote and improve health and could become a service that minimizes primary care consultations for simple cases.

This is because PU and PEOU are not the only mediators for the final intention to use. Promotion of health is directly related to behavioral intention to use. This was a highly significant relationship and means that users prefer mHealth apps that promote health. This recommendation is important for designers, developers, and start-ups creating new mHealth apps. Therefore, we could start thinking that barriers such as standards, security, and interoperability [116] could be overcome by the activities derived from promotion of health.

The significance of the association between PU and behavioral intention to use explains the importance of mHealth apps for the users. This could explain the evolution of mHealth apps that offer an increasing number of benefits to the user.

An example is that the users’ health information can now be transmitted online. This could help health centers have real-time information and minimize visits to health centers for primary care. To increase the adoption and use of mHealth apps, there should be an approved catalog of health service providers and an adoption strategy for citizens.

Based on our study results, the authorities could take the following as indicators for the use of mHealth apps: connectivity of the mHealth app, interaction between the patient and the health professional via the app, the need to prescribe additional quality hardware that allows measurements and analyses, and the personalized and nonautomated accessibility of these apps to the use and analysis of patient data remotely. These tools could be key indicators to measure the quality of this type of apps by health authorities.

In conclusion, gender is a determining factor that influences the intention to use eHealth apps, and therefore, different interfaces and utilities could be designed according to gender.

The findings of this study are beneficial for organizations, governments, and policymakers to provide strategies and policies to improve mHealth app in different hospitals and Spanish primary health care centers.

### Limitations

The limitations of the research are those related to the analysis technique used, the country under study, and the size of the sample.

## Abbreviations

PLS: partial least squares
SEM: structural equation modeling
TAM: technology acceptance model
